# Cancer-specific promoter DNA methylation of *Cysteine dioxygenase type 1 (CDO1)* gene as an important prognostic biomarker of gastric cancer

**DOI:** 10.1371/journal.pone.0214872

**Published:** 2019-04-01

**Authors:** Hiroki Harada, Kei Hosoda, Hiromitsu Moriya, Hiroaki Mieno, Akira Ema, Hideki Ushiku, Marie Washio, Nobuyuki Nishizawa, Satoru Ishii, Kazuko Yokota, Yoko Tanaka, Takeshi Kaida, Takafumi Soeno, Yoshimasa Kosaka, Masahiko Watanabe, Keishi Yamashita

**Affiliations:** 1 Department of Surgery, Kitasato University School of Medicine, Minami-ku, Sagamihara, Kanagawa, Japan; 2 Department of Breast and Endocrine Surgery, Kitasato University School of Medicine, Minami-ku, Sagamihara, Kanagawa, Japan; 3 Division of Advanced Surgical Oncology, Department of Research and Development Center for New Medical Frontiers, Kitasato University School of Medicine, Minami-ku, Sagamihara, Kanagawa, Japan; Chinese University of Hong Kong, HONG KONG

## Abstract

**Background:**

There have been few available prognostic biomarkers in gastric cancer. We rigorously assessed the clinical relevance of promoter DNA methylation of *Cysteine dioxygenase type 1* (*CDO1*) gene, a cancer-specific aberration, in human gastric cancer.

**Methods:**

Quantitative *CDO1* methylation value (TaqMeth V) was initially calculated in 138 gastric cancer patients operated in 2005, and its clinical significance was elucidated. As a subsequent expanded set, 154 gastric cancer patients with pathological stage (pStage) II / III with no postoperative therapy were validated between 2000 and 2010.

**Results:**

(1) Median TaqMeth V of *CDO1* gene methylation of gastric cancer was 25.6, ranging from 0 to 120.9. As pStage progressed, *CDO1* TaqMeth V became higher (p < 0.0001). (2) The optimal cut-off value was determined to be 32.6; gastric cancer patients with high *CDO1* gene methylation showed a significantly worse prognosis than those with low *CDO1* gene methylation (p < 0.0001). (3) A multivariate cox proportional hazards model identified high *CDO1* gene methylation (p = 0.033) as an independent prognostic factor. (4) The results were recapitulated in the expanded set in pStage III, where high *CDO1* gene methylation group had a significantly worse prognosis than low *CDO1* gene methylation group (p = 0.0065). Hematogenous metastasis was unique in pStage III with high *CDO1* gene methylation (p = 0.0075). (5) Anchorage independent growth was reduced in several gastric cancer cell lines due to forced expression of the *CDO1* gene, suggesting that abnormal *CDO1* gene expression may represent distant metastatic ability.

**Conclusions:**

Promoter DNA hypermethylation of *CDO1* gene was rigorously validated as an important prognostic biomarker in primary gastric cancer with specific stage.

## Introduction

Gastric cancer is the fifth most common malignancy and the third leading cause of cancer-related death worldwide [1]. Advanced gastric cancer, defined as depth of muscularis propria or beyond, still exhibited poor prognosis by curative surgery even in combination with effective adjuvant chemotherapy [2, 3], and require prognostic factors reflecting their biology to enrich high-risk patients for recurrences. Although several prognostic biomarkers have been reported by immunohistochemistry [4, 5] or by mRNA quantification [6, 7], they have weak points; the former included issues of intra-tumoral heterogeneity and cut-off line between positive and negative cases, while the latter is instable and not appropriate for routine examination. Hence, stable and quantitative methods have been anticipated to develop like DNA markers [8].

Epigenetic gene silencing of the tumor suppresser genes (TSGs) through promoter DNA hypermethylation is a unique feature in human cancers, whereas such cancer specific methylation is rather a rare event [8, 9]. We had developed pharmacologic reversal of epigenetic silencing and uncovered a myriad of transcriptionally repressed genes in human cancers [10–13], and have finally identified outstanding candidate TSG with frequent promoter DNA methylation, a *cysteine dioxygenase type 1* (*CDO1*) gene in human cancers including gastric cancer [14].

The CDO1 protein is a non-heme structured, iron-containing metalloenzyme involved in conversion of the cysteine to cysteine sulfinic acid (CSA), while it can promote apoptosis by increasing reactive oxygen species (ROS) through suppression of glutathione generation in breast cancer cells [15], thus suggesting that *CDO1* is a TSG in the context of pseudo-inflammatory reaction during carcinogenesis. In addition to breast cancer [15, 16], promoter DNA hypermethylation of *CDO1* gene has been reported to be highly specific to cancer cells [8], and exhibited prognostic relevance in specific cancers such as esophageal [17, 18], lung [19], colorectal [20], gallbladder [21], and kidney cancer [22].

Nevertheless, we have not found any reports on the clinical significance of *CDO1* gene methylation in primary gastric cancer. In the present study, *CDO1* gene promoter DNA methylation was for the first time examined and clarified for detailed clinicopathological factors in primary gastric cancer, and proved great clinical value in gastric cancer clinics.

## Materials and methods

### Patients and tissue samples

We recruited 140 primary gastric cancer patients who underwent curative gastrectomy at the Kitasato University Hospital in 2005. DNA was extracted from the formalin-fixed, paraffin embedded tumor tissues of the 138 patients who agreed to use pathological specimens. As an expanded set, 154 patients of pathological stage (pStage) II/III without postoperative adjuvant chemotherapy were collected from 1673 gastric cancer patients between 2000 and 2010 for validation. The median follow up term of the expanded one was 100.5 months, ranging from 2 to 148 months. pStage was used according to the Japanese Classification of Gastric Cancer staging system, 14th edition [23]. This study was approved by the Kitasato University Ethics Committee (number B17-251).

### Cell lines

The 6 gastric cancer cell lines (MKN7, Kato III, SH-10-TC, KE-97, MKN74, and NUGC-4) were previously described [24]. All cell lines were grown in RPMI-1640 medium (GIBCO, Carlsbad, CA) supplemented with 10% FBS. The hepatocellular carcinoma cell line HepG2 and colorectal DLD-1 cells were used as positive and negative controls for methylation [25].

### Genomic DNA extraction and bisulfite treatment

Genomic DNA was extracted from cell lines using the QIAamp DNA Mini Kit (Qiagen, Hilden, Germany). Formalin-fixed paraffin-embedded tissue was cut into six slices of 10 μm thick before genomic DNA extraction using the QIAamp DNA FFPE Tissue Kit (Qiagen). Genomic DNA (2 μg) was bisulfite converted using the EZ DNA Methylation-Gold Kit (Zimo Research, Irvine, CA, USA).

### Total RNA extraction and RT-PCR

Total RNA was extracted using an RNeasy Mini Kit (Qiagen). First strand cDNA was synthesized from RNA (2 μg) using SuperScript III reverse transcriptase (Invitrogen) and Oligo (dT) primers (Invitrogen). RT–PCR was carried out using Platinum Taq DNA Polymerase (Invitrogen). Primers sequences for *CDO1* and *β-actin* were previously described [21].

### Quantitative methylation-specific PCR (Q-MSP)

For Q-MSP of *CDO1* gene, we performed real-time PCR using iQ Supermix (Bio-Rad, Hercules, CA) and CFX96 real-time systems and TaqMeth V was defined as previously described [21]. All reactions were performed in triplicate.

### 5-Aza-dC and TSA treatment

Cells were seeded in a 10 cm dishes, and were then treated every 24 h for 4 days with either 1 or 5 μM 5-Aza-dC (5-aza-20-deoxycytidine) dissolved in 50% acetic acid or were mock treated with PBS including the same amount of acetic acid. Trichostatin A (TSA; 300 nM; Sigma Aldrich, Inc, St Louis, MO, USA) was added to the medium for the final 24 h. On day 5, the cells were harvested and mRNA was extracted.

### Immunostaining for CDO1 in primary gastric cancer tissues

FFPE tissue blocks were cut into thin sections (4 μm thick) and immunohistochemistry was performed as previously described [21]. The sections were incubated with primary rabbit anti-CDO1 polyclonal antibody (12589-1-AP) (proteintech, Rosemont, IL; 1:100). The secondary antibody reaction was performed using the Histofine Simple Stain MAX-PO (MULTI) kit (Nichirei, Tokyo, Japan). Mayer's Hematoxylin Solution was used to stain nuclei.

### Plasmid construction for transfection into cell lines

Full-length *CDO1* cDNA was inserted into the pcDNA3.1 myc-His C expression vector (Invitrogen) as previously described [20]. Cells were transfected using Lipofectamine 2000 (Invitrogen) in Opti-MEM (Invitrogen).

### Western blotting analysis

Total cellular protein (60 μg) was loaded onto a NuPAGE 4–12% Bis-Tris gel (Invitrogen) and electrophoresis was performed, followed by electroblotting to a PVDF membrane (Invitrogen). The blots were incubated with anti-myc (Invitrogen) and anti β-Actin (Invitrogen) antibodies as previously described [26]. Signals were detected using the luminescent image analyzer ImageQuant LAS 4000 (GE Healthcare, CT, USA).

### Cell proliferation assay

Cell proliferation was assayed using the CytoSelect water-soluble tetrazolium salt (WST-1) Cell Proliferation Assay Reagent (Cell Biolabs, San Diego, CA, USA). On day 1, the cells were cultured in a 96-well plate at a density of 1×10^4^ cells per plate. On day 2, the cells were transiently transfected with *CDO1*. On day 3, cell proliferation was evaluated by measuring the optical density (OD) at 450 nm.

### Anchorage-independent colony formation assay

The anchorage-independent colony formation assay was performed. In a six-well plate, 0.72% agarose (Bacto Agar; Becton, Dickinson and Company, Franklin Lakes, NJ) was placed on the bottom. Top agar was made with agarose mixed with 1 × 10^5^ cells transfected with *CDO1*. After 3 weeks of culture, colonies with more than 100 cells were counted in 10 fields of view. The experiment was conducted twice.

### Statistical analyses

All statistical analyses were performed using JMP 11 software (SAS Institute Inc., Cary, NC, USA). Continuous variables were evaluated by ANOVA, Student’s t test; categorical variables were evaluated by Fisher’s exact test or the Chi-square test, as appropriate. Overall survival (OS) was measured from the date of death or censored at the date of the last follow-up evaluation. Survival was estimated using the Kaplan-Meier method and compared by the log-rank test. Differences between results of comparative tests were considered significant if the two-sided P value was less than 0.05.

## Results

### Quantification of promoter DNA methylation of *CDO1* gene in primary gastric cancer

**Fig 1:**
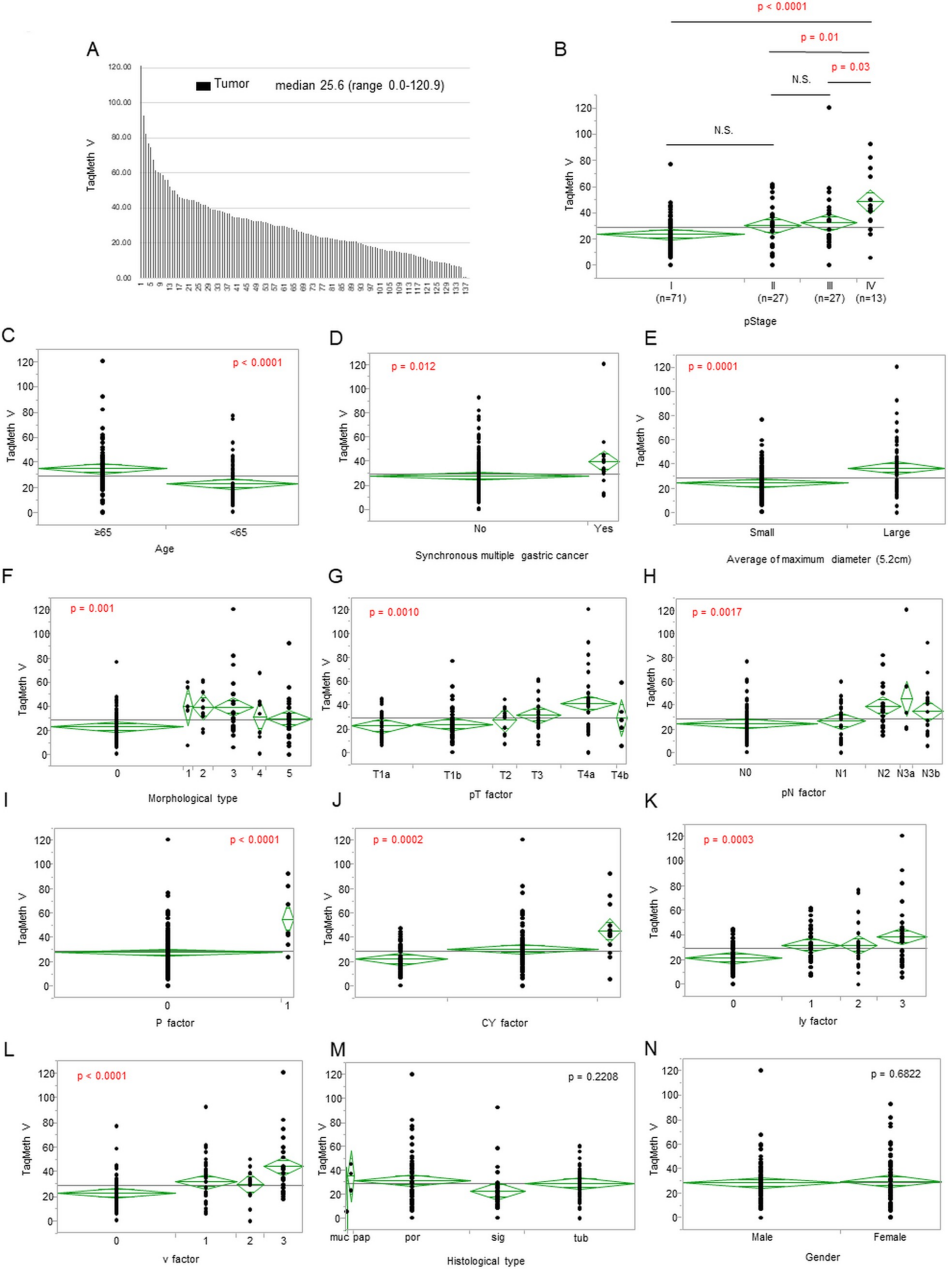
*CDO1* TaqMeth V in primary gastric cancer and its correlation with clinicopathological factor. (A) *CDO1* TaqMeth V distribution in the gastric cancer tissues. Median TaqMeth V of *CDO1* gene was 25.6, ranging from 0 to 120.9. *CDO1* TaqMeth V distribution in the gastric cancer tissues according to (B) pStage IV and pStage I / II / III (p < 0.0001 / p = 0.01 / p = 0.03), (C) Age (p < 0.0001), (D) synchronous multiple gastric cancer (p = 0.012), (E) average of maximum diameter (p = 0.0001), (F) morphological type (p = 0.001), (G) pT factor (p = 0.001), (H) pN factor (p = 0.0017), (I) P factor (p < 0.0001), (J) CY factor (p = 0.0002), (K) ly factor (p = 0.0003), (L) v factor (p < 0.0001), (M) histological type (p = 0.2208), (N) Gender (p = 0.6822).

Q-MSP for *CDO1* gene was initially performed in 138 primary gastric cancer. Median TaqMeth V of *CDO1* gene was 25.6, ranging from 0 to 120.9 (Fig 1A). The *CDO1* TaqMeth V tended to become higher as pStage progressed. There was a significant difference between pStage IV and pStage I / II / III (p < 0.0001 / p = 0.01 / p = 0.03, respectively)(Fig 1B). Age (Fig 1C, p < 0.0001), synchronous multiple gastric cancer (Fig 1D, p = 0.012), tumor size (divided by 5.2cm) (Fig 1E, p = 0.0001), morphological type (Fig 1F, p = 0.001), pT factor (Fig 1G, p = 0.001), pN factor (Fig 1H, p = 0.0017), P factor (Fig 1I, p < 0.0001), CY factor (p = 0.0002, Fig 1J), ly factor (Fig 1K, p = 0.0003), and v factor (Fig 1L, p < 0.0001) showed a significant difference. On the other hand, there was no significant difference between histological type (Fig 1M, p = 0.2208) and gender (Fig 1N, p = 0.6822).

### Prognostic analysis according to *CDO1* gene TaqMeth V in primary gastric cancer

**Fig 2:**
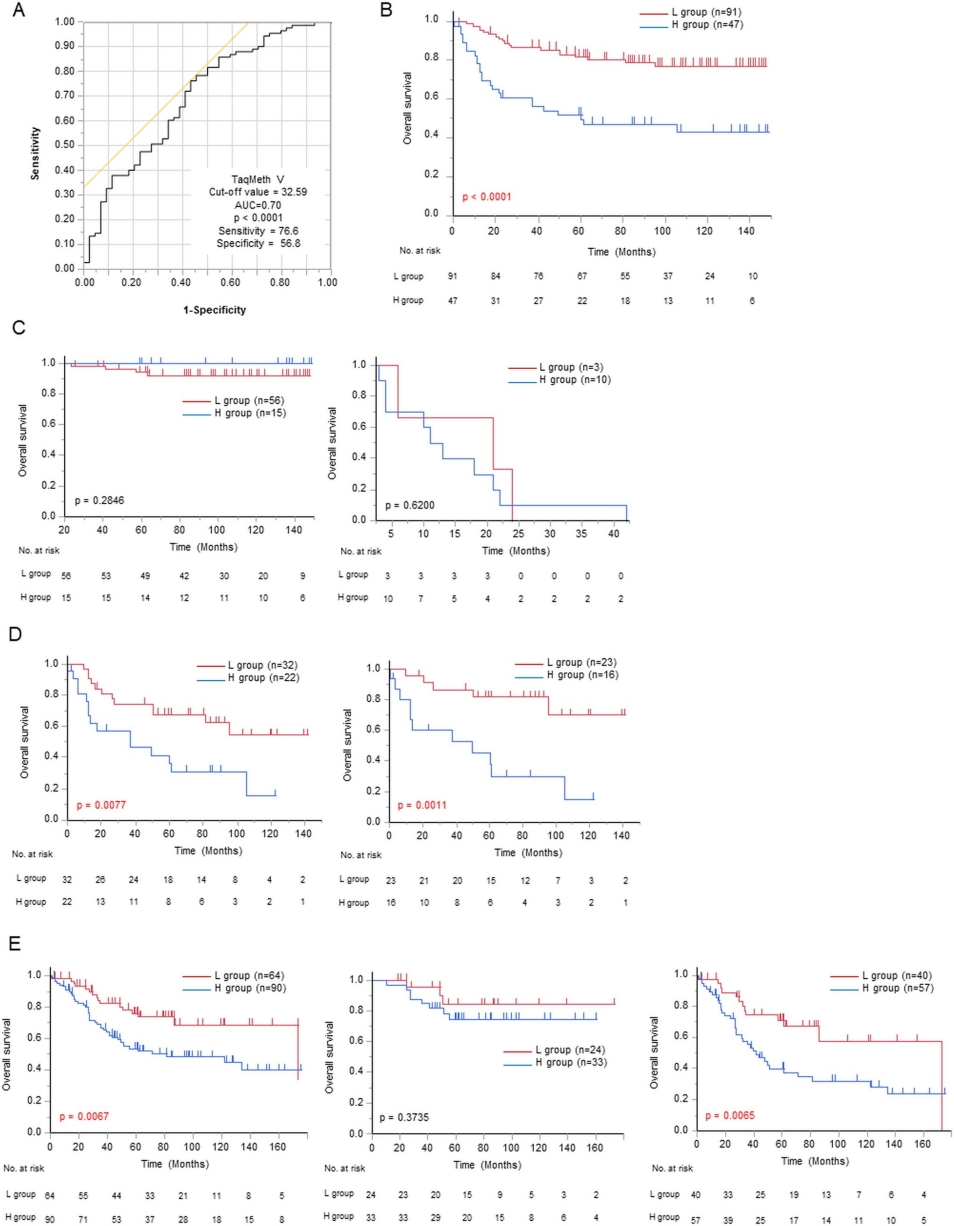
Prognostic analysis of *CDO1* TaqMeth V in primary gastric cancer. (A) ROC curve of the optimal cutoff value of *CDO1*TaqMeth V in death event. (B) Kaplan-Meier survival curves for OS comparing gastric cancer patients with *CDO1* TaqMeth V below 32.6 and those with *CDO1* TaqMeth V equal to or over 32.6 (p < 0.0001). In the same set of gastric cancer patients, survival curves are shown according to (Left panel of Fig 2C) pStage I (p = 0.2846), (Right panel of Fig 2C) pStage IV (p = 0.62), (Left panel of Fig 2D) pStage II / III (p = 0.0077). (Right panel of Fig 2D) Kaplan-Meier survival curves for OS comparing pStage II / III gastric cancer patients without postoperative adjuvant chemotherapy with *CDO1* TaqMeth V below 32.6 and those with *CDO1* TaqMeth V equal to or over 32.6 (p = 0.0011). In 154 patients as an expanded set of pStage II / III gastric cancer without postoperative adjuvant chemotherapy. (Left panel of Fig 2E) Kaplan-Meier survival curves for OS comparing gastric cancer patients with *CDO1* TaqMeth V below 32.6 and those with *CDO1* TaqMeth V equal to or over 32.6 (p = 0.0067). (Middle panel of Fig 2E) In expansion set of pStage II. Kaplan-Meier survival curves for OS comparing gastric cancer patients with *CDO1* TaqMeth V below 32.6 and those with *CDO1* TaqMeth V equal to or over 32.6 (p = 0.3735). (Right panel of Fig 2E) In expansion set of pStage III. Kaplan-Meier survival curves for OS comparing gastric cancer patients with *CDO1* TaqMeth V below 32.6 and those with *CDO1* TaqMeth V equal to or over 32.6 (p = 0.0065).

For prognostic analysis, the optimal cut-off value for OS was determined by ROC curve predicting death event. The most optimized TaqMeth V was determined to be 32.59 (AUC of 0.70, p < 0.0001, sensitivity 76.6%, specificity 56.8%)(Fig 2A). Gastric cancer patients were divided into two groups of H group (n = 47): high *CDO1* TaqMeth V group (TaqMeth V ≥ 32.6) and L group (n = 91): low *CDO1* TaqMeth V group (TaqMeth V <32.6). The H group had a significantly poorer prognosis (5-year OS 49.5%) than the L group (5-year OS 82.0%) (p < 0.0001) (Fig 2B). Age, procedures of gastrectomy, lymph node dissection, radical resection, tumor location, morphological type, pStage, and *CDO1* TaqMeth V were significant (p < 0.05) prognostic factors in a univariate analysis. These univariate prognostic factors were applied to Cox proportional hazards model. As a result, pStage and *CDO1* TaqMeth V (HR 2.28, CI 1.07–4.95, p = 0.033) were finally remnant independent prognostic factors in multivariate analysis (Table 1).

**Table 1:** Univariate and multivariate prognostic analysis for overall survival.

Clinicopathological factor	Number (%)	Univariate analysis		Multivariate analysis		
		5-year OS	*p*-Value	Hazard ratio	95% CI	*p*-Value
Age (years)			0.0064			0.3479
<65	70 (50.7%)	81.3%		Reference		
≥65	68 (49.3%)	60.6%		1.41	0.69–2.99	
Gender			0.9844			
Male	84 (60.9%)	70.5%				
Female	54 (39.1%)	71.9%				
Procedure of gastrectomy			0.0005			0.5361
Distal gastrectomy	75 (54.3%)	83.8%		Reference		
Total gastrectomy	58 (42.0%)	75.0%		1.49	0.59–3.80	
Proximal gastrectomy	5 (3.7%)	54.0%		2.00	0.10–13.08	
Field of lymph node dissection			0.0315			0.1101
D1/1+ lymph node dissection	86 (62.3%)	77.5%		Reference		
D2 lymph node dissection	52 (37.7%)	60.3%		2.07	0.85–5.33	
Synchronous multiple gastric cancer			0.6854			
Absence	121 (87.7%)	75.0%				
Presence	17 (12.3%)	70.5%				
Resectability			< 0.0001			0.3759
R0	132 (95.7%)	73.5%		Reference		
R1-2	6 (4.3%)	16.7%		1.68	0.51–4.82	
Tumor location			0.0315			0.0906
Lower	27 (19.6%)	76.7%		Reference		
Middle	99 (71.7%)	73.4%		1.37	0.49–3.51	
Upper	12 (8.7%)	35.0%		2.11	0.58–8.11	
Histological type			0.06			
Differentiated type	55 (39.9%)	81.1%				
Undifferentiated type	82 (60.1%)	63.9%				
Morphological type			< 0.0001			0.383
Early cancer	70 (50.7%)	94.1%		Reference		
Advanced cancer	68 (49.3%)	46.6%		2.56	0.28–15.00	
Pathological stage (pStage)			< 0.0001			< 0.0001
pStage I	71 (51.4%)	95.6%		Reference		
pStage II	27 (19.6%)	72.2%		10.52	1.12–80.28	
pStage III	27 (19.6%)	38.1%		43.37	3.91–455.44	
pStage IV	13 (9.4%)	0%		204.91	17.61–2128.60	
*CDO1* methylation value (ROC 32.6)			< 0.0001			0.0326
<32.6	91 (65.9%)	82.0%		Reference		
≥32.6	47 (34.1%)	49.5%		2.28	1.07–4.95	

As the H group included higher pStage than the L group did (p = 0.0006, Table 2). prognosis was then compared in individual pStage. There was no significant difference between the H group and the L group in pStage I / IV (p = 0.2846/p = 0.62, respectively) (left and right panels of Fig 2C), while the H group (n = 22) exhibited significantly poor prognosis than the L Group (n = 32) in pStage II / III (p = 0.0077) (Fig 2D, left panel). In 2005, pathological stage II / III patients were recommended for postoperative adjuvant chemotherapy as a phase III clinical trial (ACTS-GC), the prognosis may have been modified by the adjuvant chemotherapy because the ACTS-GC proved prognostic efficacy [27]. We then restricted prognostic analysis to the 39 patients with pStage II / III gastric cancer without postoperative adjuvant chemotherapy, and still proved that the H group (n = 16) had a significantly poorer prognosis (5-year OS 37.7%) than the L group (n = 23, 5-year OS 81.7%) (p = 0.0011) (Fig 2D, right panel).

**Table 2:** Correlation of clinicopathologic characteristics and CDO1 methylation.

	*CDO1* methylation value				
	Low (<32.6)		High (≥32.6)		
	n = 91 (65.9%)		n = 47 (34.1%)		
Clinicopathological factors	Number	Percentage (%)	Number	Percentage (%)	*p*-Value
Age (years)					0.0004
<65	56	40.6%	14	10.1%	
≥65	35	25.4%	33	23.9%	
Gender					NS
Male	57	41.3%	27	19.6%	
Female	34	24.6%	20	14.5%	
Histological type					NS
Differentiated carcinoma	35	25.5%	20	14.6%	
Undifferentiated carcinoma	55	40.2%	27	19.7%	
Pathological T factor (pT)					0.0024
pT1a	25	18.1%	6	4.4%	
pT1b	32	23.2%	8	5.8%	
pT2	8	5.8%	4	2.9%	
pT3	13	9.4%	9	6.5%	
pT4a	10	7.3%	18	13.0%	
pT4b	3	2.2%	2	1.4%	
Pathological N factor (pN)					0.0104
pN0	57	41.2%	16	11.6%	
pN1	16	11.6%	9	6.5%	
pN2	7	5.1%	11	8.0%	
pN3a	3	2.2%	3	2.2%	
pN3b	8	5.8%	8	5.8%	
Peritoneal dissemination (P)					0.0031
P0	90	65.2%	41	29.7%	
P1	1	0.7%	6	4.4%	
Cytorogy of peritoneal lavage (CY)					0.0017
CY0	50	36.2%	28	20.3%	
CY1	3	2.2%	9	6.5%	
CYX	38	27.5%	10	7.3%	
Pathological stage (pStage)					0.0006
pStage I	56	40.6%	15	10.9%	
pStage II	17	12.3%	10	7.2%	
pStage III	15	10.9%	12	8.7%	
pStage IV	3	2.2%	10	7.2%	
Lymph duct invasion (ly)					0.001
ly0	46	33.6%	9	6.6%	
ly1	18	13.1%	15	10.9%	
ly2	14	10.2%	7	5.1%	
ly3	12	8.8%	16	11.7%	
Veinous invasion (v)					< 0.0001
v0	56	40.8%	10	7.3%	
v1	18	13.1%	16	11.7%	
v2	8	5.8%	7	5.1%	
v3	8	5.8%	14	10.2%	

NS: not significant

In order to verify the results of prognostic significance in pStage II / III gastric cancer without adjuvant chemotherapy, prognostic analysis was performed on 154 patients in pStage II / III advanced gastric cancer without adjuvant chemotherapy who were collected between 2000 to 2010 as an expanded set. The result again proved similar prognosis with the learning set. *CDO1* gene hypermethylation could predict poorer prognosis of pStage II / III gastric cancer without adjuvant chemotherapy than *CDO1* gene hypomethylation (5-year OS 53.8% / 76.3% in the H / L group, respectively) (p = 0.0067) (Fig 2E, left panel). Statistical difference of OS was not recognized in pStage II (Fig 2E, middle panel), but in pStage III (Fig 2E, right panel); the 5-year OS of the H group was 40.1%, while 71.3% in the L group, and the prognosis was significantly poorer in the H group as compared with that in the L group (p = 0.0065).

The recurrence pattern of pStage II/III gastric cancer patients with no adjuvant chemotherapy in the expanded set was then clarified to explain the cause of poor prognosis by *CDO1* gene hypermethylation. There were 8 recurrent cases (14.0%) in pStage II and 43 recurrent cases (44.3%) in pStage III, with significant more relapses in pStage III cases than in pStage II cases (p < 0.0001). There were 15 cases (9.6%) of initial recurrences at lymph nodes, 25 cases (15.9%) of initial recurrences at distant organ, 22 cases (14.0%) of initial recurrence at peritoneum, and 3 cases of initial recurrences at local location (1.9%). First of all, there were significantly more recurrences in the H group than in the L group among the pStage III gastric cancer patients (p = 0.0052), and the most outstanding features were characterized by more distant organ metastasis in the H group than in the L group in pStage III (p = 0.0075) (Table 3).

**Table 3:** Patterns of recurrence after gastrectomy.

	Pathological stage II				Pathological stage III			
Recurrences	Low n = 24	High n = 33	Total n = 57	*P*-value	Low n = 40	High n = 57	Total n = 97	*P*-value
Total	3	5	8 (14.0%)	NS	11	32	43 (44.3%)	0.0052
Lymph node recurrence (n = 15, 9.6%)	0	3	3 (5.3%)	NS	4	8	12 (12.4%)	NS
Regional	0	0	0		2	2	4 (4.1%)	
Extra regional	0	3	3 (5.3%)		2	6	8 (8.2%)	
Hematogeneous recurrence (n = 25, 15.9%)	1	3	4 (7.0%)	NS	3	18	21 (21.6%)	0.0075
Liver	1	2	3 (5.3%)		1	9	10 (10.3%)	
Bone	0	0	0		1	4	5 (5.2%)	
Lung	0	0	0		1	1	2 (2.1%)	
Ovary	0	0	0		0	2	2 (2.1%)	
Brain	0	0	0		0	1	1 (1.0%)	
Colon	0	1	1 (1.8%)		0	0	0	
Liver+Lung	0	0	0		0	1	1 (1.0%)	
Peritoneal dissemination recurrence (n = 22, 14.0%)	2	2	4 (7.0%)	NS	6	12	18 (18.0%)	NS
Local recurrence (n = 3, 1.9%)	0	0	0	NS	0	3	3 (3.0%)	NS

NS: not significant

### Functional assessment of *CDO1* gene transfection on gastric cancer cells

**Fig 3:**
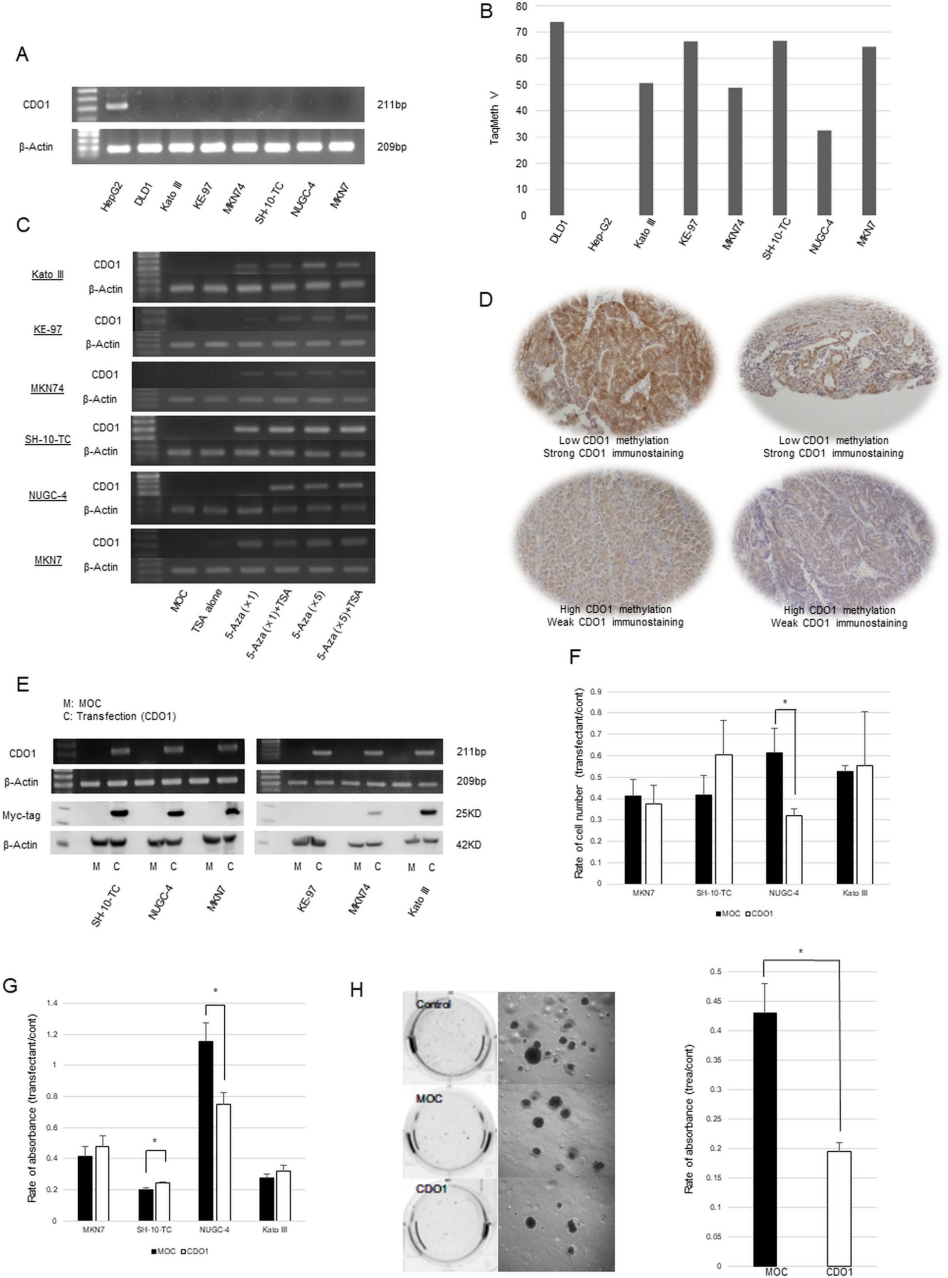
(A) Expression of *CDO1* gene in all 6cell lines in mRNA level. (B) DNA methylation levels in all 6 cell lines by using Q-MSP after bisulfite treatment. (C) Reactivation of *CDO1* gene by demethylation. In Immunostaining chemistry, (Upper panel of Fig 3D) two specimens of H group were weakly positive, and (Lower panel of Fig 3D) two specimens of L group were strongly positive. (E) *CDO1* gene was transfected to all 6 gastric cancer cell lines and the expression was confirmed by RT-PCR and Western blotting. (F) Cell count after gastric cancer cell lines was transfected *CDO1* gene. (G) Cell viability after gastric cancer cell lines was transfected CDO1 gene (WST-1 assay). (H) Confirmation of colony formation on Soft agar. When the *CDO1* gene was transfected, the number of colonies decreased compared to control and MOC. Anchorage independent growth ability significantly reduced the colony forming ability in CDO1-transfected cells in Kato III compared to MOC-transfected cells (p = 0.0245).

Expression of *CDO1* gene was never observed in 6 gastric cancer cell lines, as compared with positive expression control of HepG2 cells at mRNA level (Fig 3A), where DNA hypermethylation was confirmed in all the 6 cell lines and DNA hypomethylation was seen in HepG2 cells (Fig 3B). Reactivation by demethylation treatments using 5-Aza-dC and Trichostatin A was confirmed in all 6 the cell lines (Fig 3C), suggesting that expression of *CDO1* gene must be suppressed by epigenetic manners such as promoter DNA methylation. Immunostaining for CDO1 protein confirmed its localization in the cytoplasm of non-cancerous gastric mucosa gland cells (data not shown) or cancer cells harboring low value of *CDO1* gene methylation (L group) (Fig 3D, upper panels). On the other hand, representative specimens of H group were weakly positive for CDO1 protein expression (Fig 3D, lower panels).

The *CDO1* full length vector was transfected to all 6 gastric cancer cell lines and the expression was confirmed by RT-PCR and Western blotting (Fig 3E). Intense expression of CDO1 protein was confirmed in MKN7, SH-10-TC, NUGC-4 and Kato III, but not in MKN74 and KE-97 (Fig 3E). After *CDO1* gene transfection, cell proliferation was suppressed in NUGC-4 by simple cell count (Fig 3F, p = 0.026) and WST-1 cell viability assays (Fig 3G, p = 0.03). Among gastric cancer cell lines tested in this study, only Kato III exhibited anchorage-independent colony formation. For demonstrating the tumor suppressive activity of *CDO1* gene, only Kato III cells were considered to be appropriate. The *CDO1*-transfected cells showed suppressed capacity of anchorage independent growth compared to MOC-transfected cells (p = 0.0245) in Kato III cells (Fig 3H).

## Discussion

Promoter DNA of the *CDO1* gene is frequently hypermethylated in human cancers including gastric cancer [14], which showed the highest AUC (0.95) to differentiate tumor tissues from the corresponding non-cancerous tissues [8]. Hypermethylation in tumor tissues beyond 60% was designated as highly relevant methylation gene (HRMG), and *CDO1* gene is the most common HRMG among human cancers [8]. Using the best optimized cut-off value of TaqMeth V to discriminate tumor from non-cancerous tissues, *CDO1* gene hypermethylation is found in 72~91% in various cancers [14, 18, 21, 28]. These frequencies were determined, based on comparison of tumor tissues to the corresponding non-cancerous tissues, and affected by the methylation level of non-cancerous tissues. For example, corresponding non-cancerous tissues were relatively highly methylated for *CDO1* gene in gallbladder cancer, and threshold cut-off value became high and the frequencies were underestimated [21]. Actual methylation frequencies are therefore considered higher than the report (~90% in almost human cancer). Anyway, *CDO1* gene has an outstanding feature with regard to cancer-specific methylation in human cancer.

The current study is the first report describing clinicopathological relevance of *CDO1* gene promoter DNA methylation status in primary gastric cancer. Recent literatures of the significant association between *CDO1* methylation and poor prognosis have been reported in breast [15, 16], esophageal [17, 18], renal cells carcinoma [22], HPV associated malignancies [29], prostate cancer [30], gallbladder cancer [21], and colorectal cancer [20]. Nevertheless, there has never been reported with regard to prognostic relevance in primary gastric cancer.

In the present study, *CDO1* TaqMeth V was rigorously validated as prognostic factor of primary gastric cancer. The most importantly, it could still show the prognostic relevance in pStage II/III gastric cancer patients without postoperative adjuvant chemotherapy. We have to clearly recognize the difference of patients with adjuvant chemotherapy and those without it from a prognostic point of view, because recent adjuvant chemotherapy is really effective to pStage II/III advanced gastric cancer [2, 3]. In order to know the biological role of *CDO1* gene during natural clinical course, we had better not include gastric cancer with adjuvant chemotherapy. In our current study, *CDO1* gene promoter methylation definitely accumulates as disease progressed, and it was significantly associated with the initial recurrences at distant organs in pStage III gastric cancer. *CDO1* gene actually suppressed anchorage independent growth in Kato III cells, suggesting that it plays a functionally critical role in distant metastasis of gastric cancer.

The best optimized cut-off TaqMeth V with regard to prognosis was set as 32.6. This optimized cut-off value is always higher than those delineated tumor from non-cancerous tissues in various cancers [14, 17, 20, 21]. Moreover, cancer patients with *CDO1* gene hypermethylation showed suppressed expression of CDO1 protein in immunohistochemistry [20, 21]. These findings suggested that higher promoter DNA methylation status represents strong suppression of CDO1 protein expression, which may be linked to tumor aggressiveness. Almost cancer cell lines including gastric cancer did not recognize *CDO1* gene expression, and it is therefore difficult to conduct an experiment to suppress expression of *CDO1* gene by RNA interference using siRNA. Only HepG2, a liver cancer cell line expressed *CDO1* gene, and suppression of *CDO1* gene by RNA interference resulted in invasive capacity as described by Brait M [14]. Reflected by this functional experiment, *CDO1* gene promoter DNA methylation is an excellent prognostic marker, because it is DNA (that is stable in any environment), and it could be quantified by Q-MSP differently from immunohistochemistry.

Although there was no significant difference of the initial recurrences at peritoneum according to *CDO1* gene TaqMeth V in gastric cancer, it can be used as a cancer detection marker, because it is highly specific to cancer cells [14]. We recently reported the usefulness of DNA diagnosis using the *CDO1* gene methylation in DNA cytology test using the peritoneal lavage of gastric cancer [31]. DNA cytology of peritoneal lavage had higher diagnostic ability compared to the conventional cytology test of peritoneal lavage. Currently, prospective study is conducted to validate the clinical utility (UMIN000026191).

Cysteine biology has recently focused on cancer stem cell features [32]. CD44 variant interacts with xCT, a glutamate-cystine transporter, and permits intracellular increase of glutathione that protects stem cells, which is associated with inflammatory processes. Ablation of CD44 induced loss of xCT from the cell surface and suppressed tumor growth. CDO1 protein catalyzes the oxidation of cysteine to CSA [33], reducing intracellular cysteine concentration and subsequent reduction of glutathione. This molecular mechanism may be associated with tumor suppressive function of *CDO1* gene [15].

Our DNA was extracted from the formalin-fixed, paraffin embedded (FFPE) tumor tissues, and not from noninvasive biopsy. Unlike fresh frozen samples, FFPE samples were demonstrated to exhibit deterioration in the quality of RNA [34], while verification of DNA methylation analysis using FFPE specimens has been done, and the usefulness has been confirmed [35]. For this reason, methylation analysis was also carried out using FFPE samples in this study.

In conclusion, *CDO1* TaqMeth V was rigorously validated to be an important prognostic factor in primary gastric cancer. It was considered that the *CDO1* methylation together with its aberrant expression may be causatively involved in the distant metastasis, resulting in poor prognosis of gastric cancer. If it is possible to predict distant metastasis, selection of patients requiring preoperative adjuvant chemotherapy can be effectively made, and can be highly expected for precision medicine.
